# Relationship Between Distance Run Per Week, Omega-3 Index, and Arachidonic Acid (AA)/Eicosapentaenoic Acid (EPA) Ratio: An Observational Retrospective Study in Non-elite Runners

**DOI:** 10.3389/fphys.2019.00487

**Published:** 2019-04-26

**Authors:** Sergio Davinelli, Graziamaria Corbi, Stefano Righetti, Elena Casiraghi, Francesco Chiappero, Serena Martegani, Riccardo Pina, Immaculata De Vivo, Artemis P. Simopoulos, Giovanni Scapagnini

**Affiliations:** ^1^Department of Medicine and Health Sciences “V. Tiberio”, University of Molise, Campobasso, Italy; ^2^Department of Epidemiology, Harvard T.H. Chan School of Public Health, Boston, MA, United States; ^3^Department of Cardiology, San Gerardo Hospital, Monza, Italy; ^4^Equipe Enervit Srl, Scientific Research Unit of Enervit Spa, Milan, Italy; ^5^Sport Medicine Ambulatory, Varese Medical Campus, Varese, Italy; ^6^Channing Division of Network Medicine, Department of Medicine, Brigham and Women's Hospital and Harvard Medical School, Boston, MA, United States; ^7^The Center for Genetics, Nutrition and Health, Washington, DC, United States

**Keywords:** running, PUFA, whole blood spot, omega-3 index, AA/EPA ratio

## Abstract

**Background:** Tissue availability of polyunsaturated fatty acids (PUFA) depends on several factors, including dietary intake, physical exercise, genetic variation, and metabolic turnover. However, there is limited evidence whether running training activity *per se* may influence indices associated with PUFA metabolism such as Omega-3 (ω-3) index and arachidonic acid (AA; 20:4ω-6)/eicosapentaenoic acid (EPA; 20:5ω-3) ratio.

**Objective:** To examine the association between kilometers (Km) run per week and changes in ω-3 index and AA/EPA ratio.

**Methods:** We conducted a retrospective, observational, cohort study of 257 non-elite runners (mean age: 40.85 ± 12.17 years) who consumed no fatty acid supplements and provided a blood sample for analysis. The whole blood samples were collected by finger sticks, stored on absorbent filter paper, and then PUFA were quantified by gas chromatography (GC) and ω-3 index and AA/EPA ratio measured.

**Results:** In a multivariate linear regression model, a gradual decrease of the ω-3 index was observed with higher weekly running distance (β = −0.033; 95% CI −0.039 to −0.026; R^2^ = 0.447; *p* < 0.0001). We also found a progressive increase of the AA/EPA ratio in subjects who ran greater weekly distances (β = 0.092; 95% CI 0.038 to 0.146; R^2^ = 0.320; *p* = 0.001). No other significant associations were observed with other variables, including years of running training and weekly training frequency (hours/week). Finally, as expected, a significant inverse correlation between ω-3 index and AA/EPA ratio (β = −2.614; 95% CI −3.407 to −1.821; R^2^ = 0.336; *p* < 0.0001) was detected.

**Conclusions:** These findings suggest that distance running training and its weekly volume may negatively contribute to changes of the ω-3 index and AA/EPA ratio. Further studies with greater sample size will be required to replicate and extend these data.

## Introduction

The blood lipid concentration of polyunsaturated fatty acids (PUFA) have been widely used as biomarkers of intake and as surrogates of their enrichment in cellular membranes (Baylin and Campos, 2006). The fatty acids (FA) composition of the cell membrane reflects not only the dietary fat intake (Martorell et al., 2015), but is also influenced by several other factors such as genetic variants and physical activity (Nikolaidis and Mougios, 2004; Rzehak et al., 2009). Furthermore, it has been established that PUFA of whole blood (WB) and red blood cells (RBC) reflect the phospholipid (PL) PUFA composition of major organs and tissues (Rizzo et al., 2010; Fenton et al., 2016). PUFA may modulate the physical properties of biological membranes via alteration of membrane lipid composition, affecting numerous cellular events and physiological processes (Abbott et al., 2012). The metabolic adaptations that occur in long-term and intense physical training may lead to changes in FA membrane composition, particularly long-chain (LC) omega-3 (ω-3) and omega-6 (ω-6) PUFA (Tepsic et al., 2009). The dietary supplementation with ω-3 LC PUFA (e.g., eicosapentaenoic acid [EPA; 20:5ω-3] and docosahexaenoic acid [DHA; 22:6ω-3]) has been shown to decrease the production of inflammatory eicosanoids, cytokines, and reactive oxygen species (ROS) in athletes who engage in high-intensity and long-duration exercise such as marathon or triathlon competitions (Mickleborough, 2013; Santos et al., 2013). The anti-inflammatory nature of ω-3 LC PUFA has generally been attributed to the inhibitory effects of EPA on the synthesis of eicosanoids from the ω-6 LC PUFA arachidonic acid (AA; 20:4ω-6) (Siriwardhana et al., 2012). While a number of studies have assessed the efficacy of ω-3 LC PUFA supplementation on oxidative stress, muscle damage, and inflammation during exercise, only a few have evaluated the impact of intense physical activity on WB PUFA profile without nutritional intervention. The regulation and metabolism of WB PUFA composition are not clearly understood in exercise-trained individuals and conflicting results have been reported from several authors (Helge et al., 1999; Marini et al., 2011; Da Boit et al., 2017). Furthermore, it is widely acknowledged that regular physical activity induces a healthy body adaptation against elevated levels of oxidative stress and inflammatory mediators (Petersen and Pedersen, 2005; Gomez-Cabrera et al., 2008). There is increasing demand to assess essential FA status using rapid, accurate and cost-effective methods of blood analysis. To date, a small number of studies have investigated the use of finger sticks to measure PUFA composition in a drop of WB (Marangoni et al., 2004, 2007; Bailey-Hall et al., 2008). Despite some concerns raised about the reliability of this sample type, several methods assessing blood FA status from a finger stick sample of WB have been developed and validated in different clinical settings (Bell et al., 2011; Montgomery et al., 2013; Liu et al., 2014; Pupillo et al., 2016; Wilson and Madrigal, 2017). Furthermore, the data obtained from FA of WB are very closely correlated with those obtained by the standard method from RBC membrane. It should be also noticed that the simplicity of the assay is particularly useful to measure the FA status of large cohorts, minimally invasive and easily understandable to clinicians and general public. Despite growing interest in measuring ω-3 LC FA, since higher levels of the ω-3 EPA and DHA are associated with anti-inflammatory properties, there is a paucity of studies on athletes concerning PUFA status indicators such as ω-3 index and AA/EPA ratio. The ω-3 index, which is the sum of the two ω-3 LC FA EPA and DHA, expressed as a % of FA in RBC, has been used in several clinical trials as a biomarker for ω-3 LC PUFA exposure. Moreover, it has also been shown to be a reliable indicator of the ω-3 LC FA status in the human body (Sands et al., 2005; Von Schacky, 2010a; Aarsetoey et al., 2011). The AA/EPA ratio has been proposed as a biochemical marker of cardiovascular events. Recently, higher levels of AA/EPA ratio were associated with a greater risk of cardiovascular disease (CVD) as well as higher prevalence of depressive symptoms in subjects with systemic inflammation (Ninomiya et al., 2013; Takahashi et al., 2017; Shibata et al., 2018). To date, the associations between these indicators and the running performances have not been fully evaluated in runners. In particular, there are no detailed data on the dose-response relationship between distance run per week and changes of these indices. Using a cross-sectional design, we aimed to explore whether the levels of the ω-3 index, as well as of the AA/EPA ratio, were affected by running distance in a population of non-elite athletes runners.

## Materials and Methods

### Study Design

This was a retrospective evaluation of subjects recruited at the Sports Medicine - Varese Medical Campus, in Varese, Italy. The study was conducted according to the guidelines laid down in the Declaration of Helsinki, and all procedures were approved by the Ethics Committee of the Hospital of Varese (“Comitato Etico Ospedale di Circolo e Fondazione Macchi”) (Approval no. 10.2692017). Written informed consent was obtained from all subjects. This study has been registered with ISRCTN.org (ISRCTN12847156). The retrospective data analysis was performed following the dictates of the General Authorization to Process Personal Data for Scientific Research Purpose of the Italian Data Protection Authority (decree no. 196/2003). Data were collected from September 30, 2016 to December 15, 2017 and then extracted from a central database of the clinical laboratory services of the Inter-University Consortium SannioTech, Benevento, Italy.

### Participants

A total of 257 subjects were included fulfilling the criteria of the study. The inclusion criteria were as follows: (1) healthy male and female subjects aged between 18 and 77 years; (2) healthy on the basis of physical examination, cardiovascular assessments and laboratory tests; (3) Caucasian ethnicity; (4) willingness to have samples stored for future research; (5) absence of eating disorders; (6) subjects trained at least three times per week for a minimum of 12 consecutive months. Taking into account what was declared in their reports, dietary advice was given to the athletes a week before blood sampling to reflect similar distribution of carbohydrates, lipids, proteins, and fluids. None of the athletes were taking any drug, dietary supplement or following a special diet. Particular care was taken to exclude subjects who were taking ω-3 dietary supplements, fish oil, anabolic drugs, vitamins, or antioxidant supplements. Moreover, the following data on running habits were collected: years of running training, weekly training frequency (hours/week) and weekly running distance (Km/week).

### Experimental Procedures

Finger sticks were used to obtain dried whole blood spot samples. After sterilizing the participant's finger and applying the lancet, the first drop of blood was discarded and then a drop was placed onto a spot card (Whatman 903, Sanford, ME, USA) pretreated with an antioxidant blend (Oxystop, OmegaQuant, Sioux Falls, SD, USA) to prevent oxidative loss of PUFA. Blood spot cards were dried at room temperature in the dark for 2 weeks and subsequently stored in a −80°C freezer until shipment. Samples were sent in batches to a clinical laboratory (Inter-University Consortium SannioTech, Benevento, Italy) to quantify whole blood PUFA. For the analysis of whole blood FA, paper punches of dried blood were first transferred into reaction vials. FA methyl esters (FAME) were generated via boron trifluoride transesterification (12%; 45 min at 100°C), and FAME were extracted with 1 ml of hexane and analyzed by gas chromatography with a GC-2010 gas chromatograph (Shimadzu, Milano, Italy). We used the following experimental conditions: capillary column was a Supelco 2,560, length 100 mt, column 0.25 mm ^*^0.20 ID; the helium flow rate was 30 ml/min; oven temperature held at 170°C for 4 min and then increased at a rate of 5°C/min to 220°C. A known standard mixture (Supelco® FAME Mix, Supelco, Bellefonte, PA, USA) of FAME was used as a comparison to identify FA. This procedure is highly reliable to quantify whole blood FA and results in minimal PUFA degradation (Marangoni et al., 2004; Araujo et al., 2008; Johnston et al., 2013; Di Marino et al., 2018). The ω-3 index, defined as the sum of EPA and DHA content in erythrocytes as a % of the total amount of FA, was determined. In addition, the ω-6 PUFA AA was also quantified. The AA and EPA concentrations were expressed as ratio between area under-the-curve of each selected methyl-ester peak and the sum of all measured methyl-ester peaks. We analyzed FA that were <0.01% of peaks detected.

### Statistical Analysis

Continuous data were expressed as mean ± standard deviation (SD), while categorical variables were expressed as proportions (%). The Student's *t*-test was used to compare continuous data and the Chi-Square test (Spearman correlation coefficient, Wilcoxon- Mann-Whitney and Kruskal-Wallis tests) was performed to investigate differences in categorical variables. A multivariate linear regression model was used to test the relationships between covariates (i.e., age, gender, BMI, years of experience, training frequency [hours/week], running distance [Km/week]) and ω-3 index or AA/EPA ratio, and to select the best predictor variable with a backward stepwise algorithm. All tests were two sided and values of *p* < 0.05 were considered to be statistically significant. All analyses were carried out using SPSS statistics version 23 (IBM Corp., Armonk, NY, USA).

## Results

### Participants Characteristics

A population representing non-elite level distance runners was targeted for this study. Participant characteristics are displayed in Table 1. Briefly, the mean age was 40.85 ± 12.17 years; 190 (73.9%) were male, 67 (26.1%) were female, and the average BMI was 21.81 ± 2.30 kg/m2. The mean of weekly training kilometers (Km) and weekly running hours (h) was 50.77 ± 26.56 and 7.36 ± 4.17, respectively. The subjects had an average activity history of 8.63 ± 8.17 years. The WB compositions of EPA, DHA, and AA are presented in Table 2. In the overall population, the mean value of ω-3 index was 3.37 ± 1.65, whereas the mean value of AA/EPA ratio was 18.4 ± 10.24.

**Table 1:** Characteristics of subjects (*n* = 257) included in this study.

	**Study population**
Gender (M/F)	190/67
Age (years)	40.85 ± 12.17
Weight (kg)	66.11 ± 10.75
BMI (kg/m^2^)	21.81 ± 2.30
Years of experience	8.63 ± 8.17
Hours per week (h/week)	7.36 ± 4.17
Km per week (km/week)	50.77 ± 26.56

*M, male; F, female; BMI, body mass index*.

*Values are presented as mean ± SD, except for gender*.

**Table 2:** Percentage of fatty acids of interest in the whole blood of the study group.

	**Mean**
EPA (20:5ω-3)	0.61 ± 0.63
DHA (22:6ω-3)	1.85 ± 0.99
AA (20:4ω-6)	6.95 ± 1.94
ω-3 index	3.37 ± 1.65
AA/EPA	18.4 ± 10.24

*EPA, eicosapentaenoic acid; DHA,docosahexaenoic acid*.

*ω-3, omega 3; AA, arachidonic acid*.

*Values are presented as mean ± SD*.

### Effect of Running Distance on the Omega-3 Index

**Figure 1:**
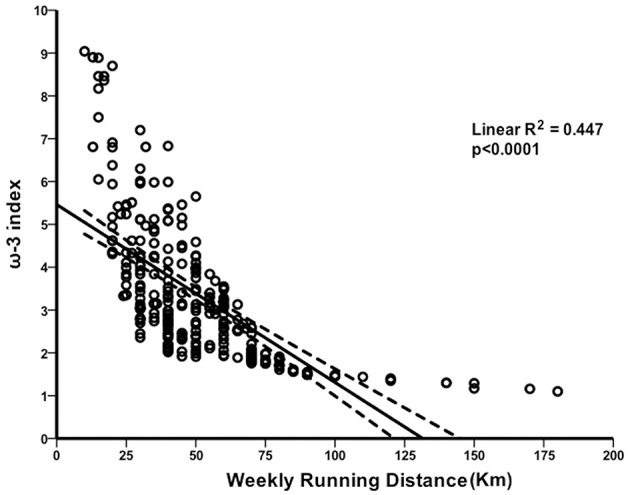
Association between the omega-3 index and distance run per week. Scatter-plot of the linear inverse relationship between omega-3 (ω-3) index and weekly running volume.

A multivariate backward stepwise analysis was performed with selected independent variables such as age, BMI, AA/EPA ratio, years of running training, weekly training frequency, and weekly running distance and using the ω-3 index as dependent variable. The multivariate regression model revealed a significant correlation between ω-3 index and Km run per week. In particular, higher weekly running distance represented the best predictor associated with low ω-3 index (β = −0.033; 95% CI −0.039 to −0.026; R^2^ = 0.447; *p* < 0.0001 (Figure 1). Thus, compared with runners who ran less, the levels of the ω-3 index decreased with the distance run per week. No other significant associations between the ω-3 index and any of the other variables were found.

### Impact of Running Distance on the AA/EPA Ratio

**Figure 2:**
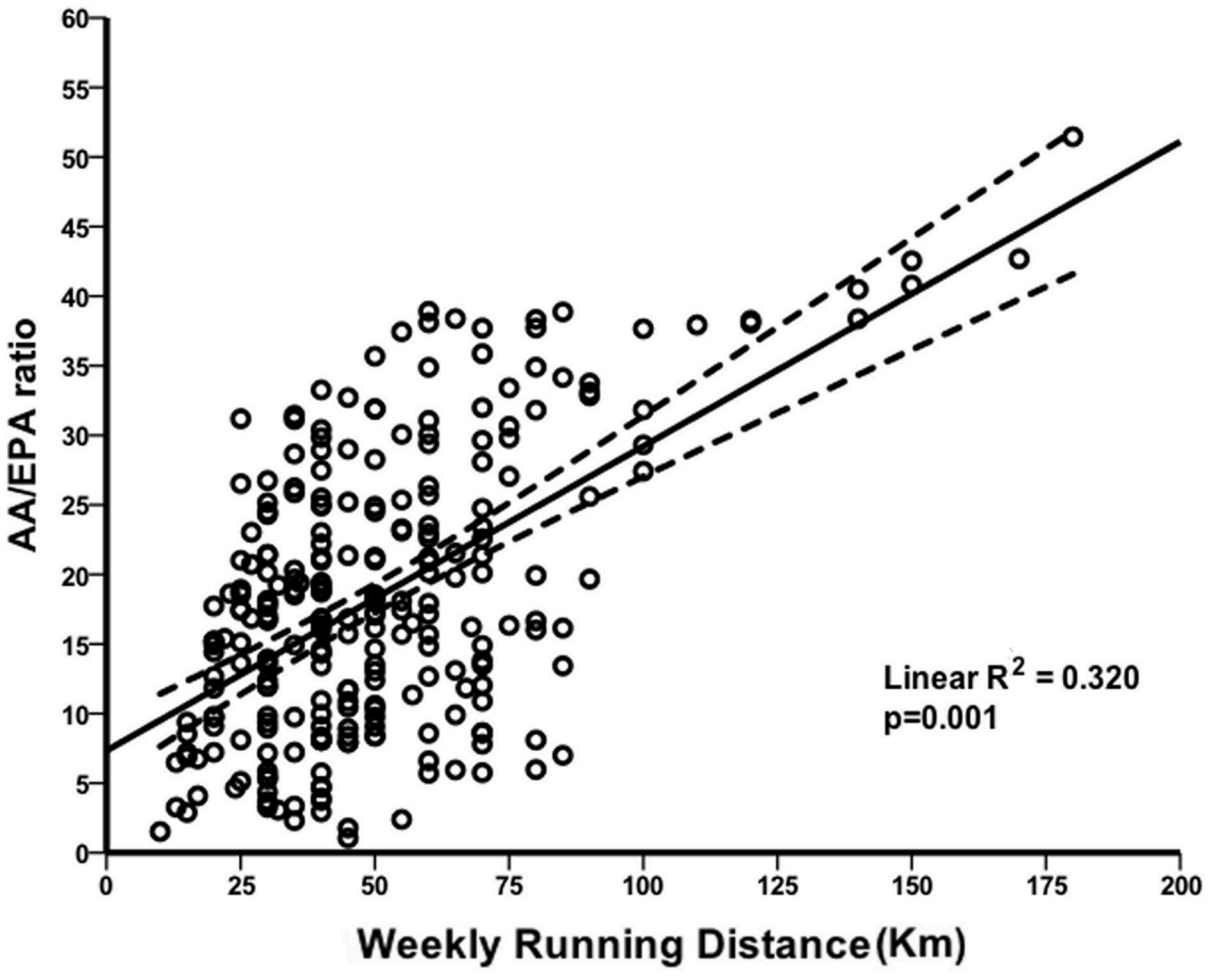
Correlation between AA/EPA ratio and distance run per week. Scatterplot of the linear direct association between arachidonic acid (AA)/eicosapentaenoic acid (EPA) ratio and weekly running distance.

In a multivariate model, the potential associations between the AA/EPA ratio and a set of predictor variables were investigated among the overall study group. We found no evidence of significant associations with age, gender, BMI, number of years of running, and weekly training frequency. However, we observed that higher weekly running distance was a good predictor variable associated with a significant increase of the AA/EPA ratio in this population of runners (β = 0.092; 95% CI 0.038 to 0.146; R^2^ = 0.320; *p* = 0.001). Figure 2 shows the progressive increment of the AA/EPA ratio in individuals who ran greater weekly distances, suggesting that the metabolism of AA/EPA in WB is closely correlated with high intensity running activity.

### Correlations Between Omega-3 Index and AA/EPA Ratio

**Figure 3:**
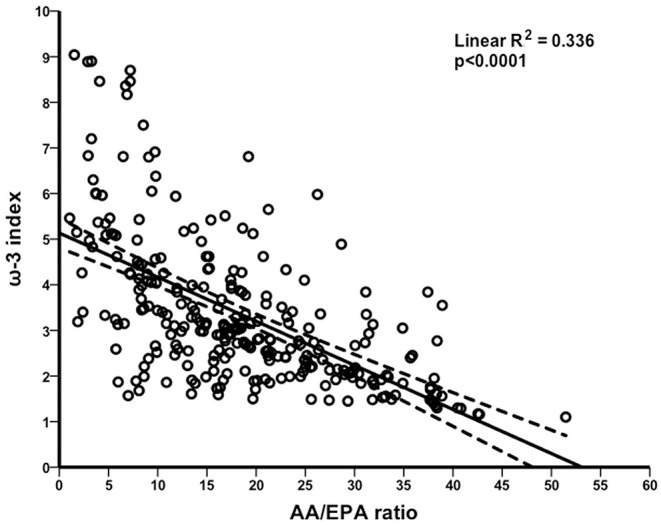
Comparison between omega-3 index and AA/EPA ratio. Scatterplot of the linear inverse correlation between omega-3 (ω-3) index and arachidonic acid (AA)/eicosapentaenoic acid (EPA) ratio in the whole blood of non-elite runners.

Because the ω-3 LC FA EPA is a shared component and an essential driver of these ratio-based metrics, we were next interested in whether ω-3 index was correlated with AA/EPA ratio. According to this analysis, the above-mentioned independent variables were included in a multivariate regression model, in which the ω-3 index as independent variable was also introduced. Statistical analysis revealed a significant inverse correlation between ω-3 index and AA/EPA ratio (β = −2.614; 95% CI −3.407 to −1.821; R^2^ = 0.336; *p* < 0.0001) in the WB of the study population. Figure 3 shows the overall decrease of the ω-3 index with AA/EPA ratio increase, suggesting that a high level of AA/EPA ratio is associated with low ω-3 index.

## Discussion

This study shows the relationships between ω-3 index, AA/EPA ratio and weekly running distance in a population of non-elite runners. To the best of our knowledge, the association between changes in these parameters and weekly running distance has not been previously shown in well-trained athletes. There are very limited data on PUFA metabolism after a moderate or an intense training, particularly in runners. Therefore, our data contribute to a better understanding of the influence of running on LC PUFA levels. The assessment of LC PUFA status using biomarkers or key FA ratios is still limited. However, clinical and epidemiological studies suggest that indices such as ω-3 index and AA/EPA ratio may provide valuable information on nutritional needs, health outcomes, and long-term disease risk (Wagner et al., 2015; Nagata et al., 2017; Davinelli et al., 2018a,b). Although several nutritional intervention trials with ω-3 FA supplements in athletes already observed changes on the FA composition of plasma, RBC, and WB (Bloomer et al., 2009; Martorell et al., 2014), the main objective of this study was to investigate whether regular running training *per se* influences two of the main biomarkers associated with PUFA metabolism. In particular, our data demonstrate that the level of ω-3 index decreases progressively with increased weekly running distance in a dose-response relationship. The observed decrease was evident at the lowest running distance and progressing in the subjects with the highest running distance. This finding indicates that regular distance running training induces changes in PUFA metabolism and should thus be considered as a modulator of PUFA tissue composition. Our results are consistent with a previously published study in athletes, which addressed a similar issue. The report by Von Schacky et al. determined the ω-3 index in 106 consecutive German elite winter endurance athletes. They found a deficiency of EPA and DHA associated with low ω-3 index (Von Schacky et al., 2014). Also a recent study by Wilson and Madrigal in collegiate athletes demonstrated that a poor intake of EPA and DHA, assessed by a food frequency questionnaire (FFQ), was the main predictor of a low ω-3 index (Wilson and Madrigal, 2016). Importantly, the population of our study may have an elevated risk for sudden cardiac death or for fatal and nonfatal myocardial infarction. Elite and non-elite athletes have an increased incidence of sudden death and a low ω-3 index has been proposed as a new risk marker and risk factor for CVD, particularly sudden cardiac death (Von Schacky, 2010b; Davinelli et al., 2014; Schmied and Borjesson, 2014; Corbi et al., 2015). Therefore, the use of this biomarker may be a potential option to prevent cardiovascular events in athletes. Additionally, our study revealed a significant inverse association between ω-3 index and AA/EPA ratio, providing a comprehensive evidence of their functional interaction. Recent data have expanded the concept that inflammation may be a critical component of myocardial dysfunction, particularly in highly trained athletes (La Gerche et al., 2015; Gabrielli et al., 2018). The ω-6 LC FA AA is the precursor of prostaglandins and leukotrienes, mediators that cause vessel inflammation and endothelial and platelets dysfunction, and has been related with CVD. On the other hand, the ω-3 LC FA EPA plays an important role to suppress the inflammatory responses by competing with AA (Simopoulos, 2008). The interaction between AA and EPA is complex and still not properly understood, however, several findings support the hypothesis that the balance between AA and EPA is important to regulate the synthesis of inflammatory mediators and maintain cardiovascular health, especially during high-intensity exercise (Simopoulos, 2007; Wada et al., 2007; Serikawa et al., 2014). Andersson et al. demonstrated that 6 weeks of low-intensity exercise training resulted in significant changes in muscle phospholipid FA composition with a significant increase in oleic acid (18:1ω9) and a decrease in AA. Although great care was taken to attempt to control dietary FA profile, the subjects were free living (Andersson et al., 1998). Helge et al. investigated the effects of regular exercise training on skeletal muscle phospholipid FA composition in humans applying a one-leg training model in which the other leg served as a control. Dietary FA composition, initial level of training, and other relevant variables were controlled. Their working hypothesis was that regular training, primarily through its effect on substrate flux and substrate storage, induces an adaptive response in muscle membrane phospholipid FA composition. Training improves insulin sensitivity, which in turn may affect performance by modulation of fuel availability. Insulin action, in turn, has been linked to specific patterns of muscle structural lipids in skeletal muscle. This study investigated whether regular exercise training exerts an effect on the muscle membrane phospholipid FA composition in humans. Seven men performed endurance training of the knee extensors of one leg for 4 weeks. The other leg served as a control. Muscle biopsies were obtained from the vastus lateralis before, after 4 days, and after 4 weeks. After 4 weeks, the phospholipid FA contents of oleic acid and DHA were significantly higher in the trained (10.9% ± 0.5% and 3.2% ± 0.4% of total FA, respectively) than the untrained leg (8.8% ± 0.5% and 2.6% ± 0.4%; p < 0.05). The ratio between ω-6 and ω-3 FA was significantly lower in the trained (11.1 ± 0.9) than the untrained leg (13.1 ± 1.2; *p* < 0.05) (Helge et al., 2001). Whereas, our results on whole blood and RBC are opposite to those of Helge, since Helge did not measure AA, EPA and DHA in the blood, but only in muscle phospholipids, and we measured them only in the blood and not in muscle phospholipids, it is quite possible that exercise training moves DHA into the muscle or proper metabolism and as such, displaces the AA from the muscle into the blood, resulting in an increase in the AA to EPA ratio. It's for this reason that it is recommended that athletes should take 1–2 grams of EPA and DHA on a daily basis and that their diet is balanced in the ω-6 and ω-3 FA. The strengths of our study are: (1) a relatively large population with a wide age range; (2) the use of FA biomarkers strictly associated with PUFA metabolism; (3) this is the first report to investigate whether the ω-3 index and AA/EPA ratio were affected by running distance. However, we acknowledge that our study has a number of limitations. The evaluation of ω-3 index and AA/EPA ratio was based on a single measurement. Although markers of inflammation, fish intake, and genetic variation of FA desaturase and elongase enzymes influence the LC PUFA status, these factors were not included in the study. As this was an observational study, our findings are not sufficient to establish a mechanistic relationship between the ω-3 index, AA/EPA ratio, and running distance. Another important limitation is the lack of a paired control group, such as untrained subjects, athletes of other sports modalities, or athletes undergoing diverse training routines, with whom the data of the runners of this study would be compared. Overall, the present analysis showed that ω-3 index and AA/EPA ratio were significantly associated with weekly running distance. These associations suggest that prolonged running activity may negatively influence PUFA profile. Therefore, future studies could assess whether regular or high intake of ω-3 rich foods may be effective to counteract the effects of running and other endurance activities on PUFA status. Further large-scale cohort studies will be needed to confirm the influence of running activity on the ω-3 index and AA/EPA ratio.

## Ethics Statement

Ethical approval was provided by the Ethics Committee of the Hospital of Varese (Comitato Etico Ospedale di Circolo e Fondazione Macchi) (Approval number. 10.2692017).

## Author Contributions

SD, GC, SR, RP, and GS were involved in the concept and design of the study. EC, FC, and SM were involved in sample collection. SD and GC were involved in writing of the manuscript. SD, GC, and SR were involved in statistical analyses. SD, GC, ID, AS, and GS were involved in interpretation of data. SD, GC, SR, RP, ID, AS, and GS were involved in critical review of the manuscript.

### Conflict of Interest Statement

EC, FC, and RP were employed by company Equipe Enervit Srl. The remaining authors declare that the research was conducted in the absence of any commercial or financial relationships that could be construed as a potential conflict of interest.
